# Investigation of systemic folate status, impact of alcohol intake and levels of DNA damage in mononuclear cells of breast cancer patients

**DOI:** 10.1038/sj.bjc.6602530

**Published:** 2005-04-05

**Authors:** M M I Hussien, H McNulty, N Armstrong, P G Johnston, R A J Spence, Y Barnett

**Affiliations:** 1 Breast Surgery Unit, Belfast City Hospital, Lisburn Road, Belfast, N Ireland BT9 7AB, UK; 2 Breast Surgery Unit, Level 3 west, Norfolk and Norwich University Hospitals, Colney Lane, Norwich, NR4 7UY, UK; 3 School of Biomedical Science, University of Ulster, Coleraine, N Ireland, UK; 4 Center for Cancer Research and Cell Biology, Queen's University, Belfast, N Ireland, UK; 5 College of Science and Technology, The Nottingham Trent University, Nottingham, England, UK

**Keywords:** folate, breast cancer, DNA damage

## Abstract

Folate is required for DNA synthesis, repair and methylation. Low folate status has been implicated in carcinogenesis, possibly as a result of higher rate of genetic damage. The aim of this study is to compare folate status and levels of DNA damage between breast cancer and benign breast disease control patients. Fasting blood samples from 64 histologically confirmed untreated breast cancer patients (mean age 57 years) and 30 benign breast disease control patients (mean age 51 years) were obtained. Red cell folate (RCF) and plasma homocysteine were measured. Mononuclear cells (MNC) were isolated for genetic damage analysis using the basic alkaline comet assay. Results are expressed as tail moment. Data were log transformed as appropriate before analysis for normalisation purposes. The geometric mean (95% confidence interval) of RCF (ng ml^−1^) in breast cancer patients was 339.07 (333.3–404.6) *vs* 379.5 (335.8–505.2) in control patients (*P*=0.24). Corresponding plasma homocysteine concentrations (*μ*mol l^−1^) were 11.9 (10.6–16.4) *vs* 10.1 (9.3–11.9) (*P*=0.073), respectively. The mean tail moment (s.d.) of DNA damage in MNC of breast cancer patients detected by the basic comet assay was 1.4 (0.66) *vs* –0.17 (0.79) in controls (*P*<0.0001, *t*-test), the modified comet assay ‘endonuclease III (Endo III)’ was 1.7 (0.70) *vs* 0.86 (0.81) (*P*<0.0001, *t*-test), and the modified comet assay ‘formamidopyrimidine glycosylase (FPG)’ was 1.6 (0.62) *vs* 0.99 (0.94) (*P*<0.0001, *t*-test). There was a significant negative correlation between RCF levels and DNA damage detected by modified comet assay ‘FPG’ (Pearson Correlation Coefficient *r*^2^=−0.26, *P*=0.02) and DNA damage was found to be significantly higher in MNC of breast cancer patients compared to benign breast disease control patients. Breast cancer patients tended to have lower RCF levels and higher levels of plasma homocysteine, but these differences were not significant. The study provides preliminary evidence that reduced folate status may be implicated in the aetiology of breast cancer perhaps by increasing the *in vivo* level of genetic instability.

Breast cancer remains one of the most common solid epithelial neoplasms. In the UK, there is an estimated annual incidence of 20 000 new cases per year, with a worldwide incidence of one million new cases per year (Sainsbury, 1999). Despite the fall in mortality in recent years, the incidence of breast cancer in the UK is rising (Sainsbury, 1999).

Collectively, the evidence from epidemiological, animal and human studies strongly suggests that folate status modulates the risk of developing cancers in selected tissue, the most notable of which is the colorectum (Kim, 1999). Folate depletion appears to enhance carcinogenesis, whereas folate supplementation may convey a protective effect (Kim, 1999). Recent studies suggest that folate may play an important role in the prevention of breast cancer, particularly among women consuming alcohol (Zhang, 2004). The means by which this modulation of cancer risk is mediated is not known with certainty, but there are several possible mechanisms (Zhang, 2004). Folate plays an integral role in DNA synthesis and methylation (Rampersaud *et al*, 2002). It is believed that folate deficiency affects DNA stability through two potential pathways. 5,10-Methylenetetrahydrofolate donates methyl group to uracil, converting it to thymine, which is used for DNA synthesis and repair. If folate is limited, imbalances in the DNA precursors occur, and uracil may be misincorporated into DNA. Subsequent misincorporation and repair may lead to double-strand breaks, chromosomal damage and cancer (Duthie *et al*, 2002). Folate affects gene expression by regulating cellular *S*-adenosylmethionine (SMA) levels. 5-Methyltetrahydrofolate is the methyl donor in the remethylation process of homocysteine to methionine, which is converted to SAM. SAM methylates specific cytosines in DNA and thus regulates gene transcription. As a consequence of folate deficiency, cellular SAM is depleted, which in turn induces DNA hypomethylation and potentially induces proto-oncogene expression leading to cancer (Duthie *et al*, 2002).

Epidemiological evidence relating folate intake to breast cancer is limited. Some case–control studies reported an inverse association between dietary folate intake and breast cancer risk (Graham *et al*, 1991; Freudenheim *et al*, 1996; Levi *et al*, 2001). Graham *et al* (1991) reported 30% lower risk of breast cancer among postmenopausal women who consume higher intake of dietary folate. In another case–control study, premenopausal women who consumed at least 460 *μ*g day^−1^ of folate had a 50% lower risk of breast cancer than women who consumed 304 *μ*g day^−1^ or less (Freudenheim *et al*, 1996). A Swiss case–control study also reported a significant inverse association between folate intake and risk of breast cancer in postmenopausal women, and this association was stronger in women who consumed alcohol (Levi *et al*, 2001). The population-based Shanghai Breast Cancer Study evaluated the association of dietary folate intake and breast cancer risk in China between 1996 and 1998 among 25–64 year old women, who never drank alcohol regularly or used vitamin supplement (Shrubsole *et al*, 2001). Dietary folate intake was significantly inversely associated with breast cancer risk in women who were in the highest (320 *μ*g day^−1^) *vs* lowest (214 *μ*g day^−1^) quartile of folate intake (Shrubsole *et al*, 2001). On the other hand, some case–control studies have failed to show any protective effect of folate against breast cancer (Thorand *et al*, 1998; Potischman *et al*, 1999; Negri *et al*, 2000). One recently published study reported that higher intakes of folate in early adult life does not reduce the risk of breast cancer in premenopausal women (Cho *et al*, 2003).

Despite extensive investigation of the association between alcohol consumption and breast cancer risk, a concordance of opinion is not apparent (Kropp *et al*, 2001). Moreover, there is evidence from two large epidemiological studies that folate status may be a factor which affects the association between alcohol intake and breast cancer risk (Zhang *et al*, 1999; Rohan *et al*, 2000). In the meta-analysis conducted by Longnecker *et al* (1988), there was a strong evidence to support a dose–response relation between breast cancer risk and alcohol intake. A pooled analysis of six prospective cohort studies conducted in Canada, the Netherlands, Sweden and the US, which included 322 ,647 women with 4335 cases of breast cancer diagnosed during the 11 years of follow-up period, reported results which were not clearly supportive that alcohol consumption is associated with breast cancer incidence (Smith-Warner *et al*, 1998).

Although there is some evidence to link lower folate to a higher risk of breast cancer, the evidence is not entirely consistent and most reported studies evaluated folate only on the basis of dietary intake data. The aim of this study was to compare the folate status measured by red cell folate (RCF) concentrations in breast cancer and control patients, and to relate this to levels of DNA damage in both groups.

## PATIENT RECRUITMENT

Ethical approval for this cross-sectional study was granted by the Research Ethical Committee of Queen's University of Belfast. All patients gave written informed consent. Patients were recruited from the Breast Surgery Unit, Belfast City Hospital between August 2000 and August 2001. All patients undergoing surgery in the Breast Surgery Unit during the study period (300 breast cancer patients) were screened for potential inclusion in the study. The diagnosis of breast cancer and benign breast disease were histologically confirmed. Benign breast disease patients required surgery either at their own request or due to repeated episodes of inflammation around the nipple and areola (duct ectasia). A total of 64 pre- and postmenopausal breast cancer patients (cases) and 30 benign breast disease patients (controls) were recruited. All patients and controls were recruited within 3 weeks of the histological diagnosis. All patients were asked to complete a short questionnaire including past medical history, drug history and basic lifestyle questionnaire that included details about alcohol consumption and smoking.

The inclusion criteria were histologically confirmed pre- and postmenopausal breast cancer patients prior to any treatment and histologically confirmed pre- and postmenopausal benign breast disease patients.

Exclusion criteria were patients taking any vitamin supplements, or those with gastrointestinal disease (including inflammatory bowel disease, malabsorption, coeliac disease) and patients with previous gastric or intestinal surgery. Patients taking medications known to interfere with folate metabolism, for example, antiepileptics, oral contraceptive pills and sulfasalazine, were also excluded. In addition, we excluded patients with ductal carcinoma *in situ* (DCIS) and patients with benign breast diseases that are known to increase the risk of breast cancer including ductal or lobualr epithelial hyperplasia.

## LABORATORY METHODS

A fasting blood sample (30 ml) was collected in the early morning before surgery for subsequent analysis of folate status (15 ml EDTA tube) and for isolation of mononuclear cells (MNC) (15 ml lithium heparin-coated tube) for DNA damage analysis. The patients then underwent surgery. A RCF lysate was prepared by diluting blood 1 : 10 with freshly prepared 1% ascorbic acid solution, wrapped in foil and mixed for 30 min, then stored at −80°C. Full blood picture analysis, including packed cell volume (required for the calculation of RCF concentration, that is, RCF=whole blood folate divided by packed cell volume) was measured in the remaining whole blood using an automated counter in Belfast City Hospital Trust Laboratories. All samples were stored at −80°C for batch analysis at the end of the study.

Mononuclear cells were separated within an hour of blood sample collection. The cell pellet was suspended in 1 ml the Hanks Balanced Salt Solution (HBSS) (Gibco, UK) and the cells were counted using a haemocytometer or by automatic cell counter to ensure a concentration of 2–3 × 10^6^ cells ml^−1^. Cell viability was checked using trypan blue (which stains dead cells a deep blue colour) to ensure viability of 80–90%. The cells were mixed with a freeze down medium (1.3 ml HBSS, 0.2 ml dimethyl sulphoxide and 0.56 ml autologous serum). This solution was transferred to −86°C freezer and subsequently into liquid nitrogen after 24 h for long-term storage.

The relationship between DNA damage markers and folate status was examined in both cases and controls by analysis of the blood samples for RCF levels using the microbiological assay (Molloy and Scott, 1997) and plasma homocysteine levels using the immunoassay (Leino, 1999). DNA damage biomarkers were measured in the MNC using the alkaline comet assay (Singh *et al*, 1988) and the modified comet assay (Collins *et al*, 1993) as described below.

### Measurement of DNA damage: single cell gel electrophoresis (comet) assay

Levels of DNA damage (DNA single-strand breaks and alkali-labile lesions) in T-cell clones were determined using the alkaline comet assay, according to the method of Singh *et al* (1988), and also the modified alkaline comet assay described by Collins *et al* (1993).

In the modified comet assays, T cells embedded on slides were treated with either formamidopyrimidine glycosylase (FPG), which recognises oxidatively modified purines (Boiteux *et al*, 1992), or with endonuclease III (ENDO III), which recognises oxidatively modified pyrimidines (Asahara *et al*, 1989). These enzymes nick DNA at the sites of oxidatively damaged nucleotides, creating single-strand breaks which can be detected with the alkaline comet assay. T cells treated with 150 mM hydrogen peroxide for 5 min at 4°C (to induce oxidative DNA damage) were used as internal positive controls in the modified alkaline comet assay to verify enzyme activity.

The comet assays were performed at 4°C to minimise the repair of existing basal levels of DNA damage present in the T cells. Cells were embedded in a 1% agarose gel on frosted microscope slides (2 × 10^4^ cells gel^−1^), and lysed for at least 1 h in a high salt alkaline buffer (2.5 M NaCl, 0.1 M EDTA, 0.01 M Tris, 1%(v v^−1^) Triton X-100, pH 10). For the modified comet assay, slides were then equilibrated in enzyme buffer (0.04 M HEPES, 0.1 M KCl, 0.5 mM EDTA, 0.2 mg ml^−1^ BSA, pH 8.0) prior to application of FPG or ENDO III. Slides treated with the lesion-specific enzymes were incubated at 37°C in a humid dark chamber for 45 min. Following enzyme treatment (or directly after alkaline lysis in the case of the alkaline comet assay), the slides were placed in electrophoresis buffer (0.3 M NaOH, 1 mM EDTA, pH 13) for 40 min. This period of incubation is to allow unwinding of DNA to be initiated from strand breaks, and then electrophoresis current is applied at 25 V, 300 mA, for 30 min. Following electrophoresis, the slides were neutralised using 0.4 M Tris pH 7.5 and stained with 50 *μ*l of 20 *μ*g ml^−1^ ethidium bromide. Stained slides were digitally analysed using UV microscopy and Komet 3.0 analysis software (Kinetic Imaging, UK), counting 50 cells per slide. DNA damage results were expressed as percentage DNA in the comet tail.

#### Internal controls

An established stable cell line was used as an internal control for all comet assays. The cells are lymphoblastoid human T-lymphocyte cells, RJK 853 clones (Yang *et al*, 1984; Beare *et al*, 1993). These internal control cells were used to demonstrate the reproducibility of the technique, and to calculate the coefficient of variation (CV) of each experiment (CV=standard deviation/mean).

### Statistics

All statistical analysis was performed using the statistics package for the Social Sciences (SPSS) version 10 computer software package (Chersey, UK). For all statistical tests, *P*-values <0.05 were considered significant. Plasma homocysteine, RCF and DNA damage (tail moment) data were skewed and were log transformed to normalise the data for comparison (which was subsequently performed using the *t*-test). Correlation analysis was performed at the log scale using the Pearson Correlation.

## RESULTS

Although breast cancer and control patients were not singularly matched, their distribution was homogenous and no adjustment for anthropometric measurements, age or menopausal status was needed (Table 1). All patients had no high-risk family history of breast cancer. A total of 42 patients did not consume alcohol (26 cancer patients and 16 controls) (Table 1).

All patients recruited for the control group had histologically confirmed benign breast conditions. There were 13 fibroadenomata, nine benign inflammatory lesions, four breast reductions/nipple eversions, two lipomata and two sebaceous cysts of the breast. Patients with a fibroadenoma had a surgical excision due to their own request. Inflammatory lesions of the breast were all due to mammary duct ectasia. The postoperative histopathology of all surgically removed specimens confirmed benign breast disease with no evidence of cellular atypia or hyperplasia.

The mean value (95% confidence interval) of RCF of breast cancer patients was 369.0 (333.3–404.6) ng ml^−1^ compared to 420.5 (335.8–505.2) ng ml^−1^ for control patients. As the RCF values were skewed (positively skewed in control patients towards higher values, and negatively skewed in breast cancer patients towards lower values), all values were log transformed to normalise the data for comparison. Red cell folate levels for breast cancer patients and controls are shown in Table 2.

The mean value (95% confidence interval) of plasma homocysteine of breast cancer patients was 13.5 (10.6–16.4) *μ*mol l^−1^ compared to 10.6 (9.3–11.9) *μ*mol l^−1^ for control patients. The plasma homocysteine values were also skewed, and were log transformed to normalise the data for comparison. Results were presented as geometric mean (95% confidence interval of the geometric mean) and compared by using the *t*-test (Table 2). Although the results were not statistically significant, breast cancer patients tended to have lower RCF and higher plasma homocysteine levels than control patients.

### DNA damage analysis

Following the basic alkaline comet assay, the mean (s.d.) tail moment for breast cancer patients was 5.0 (3.4) *vs* 1.1 (1.2) for control patients. The mean (s.d.) tail moment detected by the modified comet assay using Endonuclease III (which detects additionally oxidised pyrimidins) for breast cancer patients was 7.5 (6.2) *vs* 3.1 (2.3) for control patients. The mean (s.d.) tail moment detected by the modified comet assay using formamidopyrimidine glycosylase ‘FPG’ (which detects additionally oxidised purines) for breast cancer patients was 6.3 (3.6) *vs* 3.7 (2.7) for control patients.

The tail moment values were highly positively skewed and for the purpose of normalisation, these were log transformed. The data were presented as log mean tail moment (Table 3).

**Figure 1:**
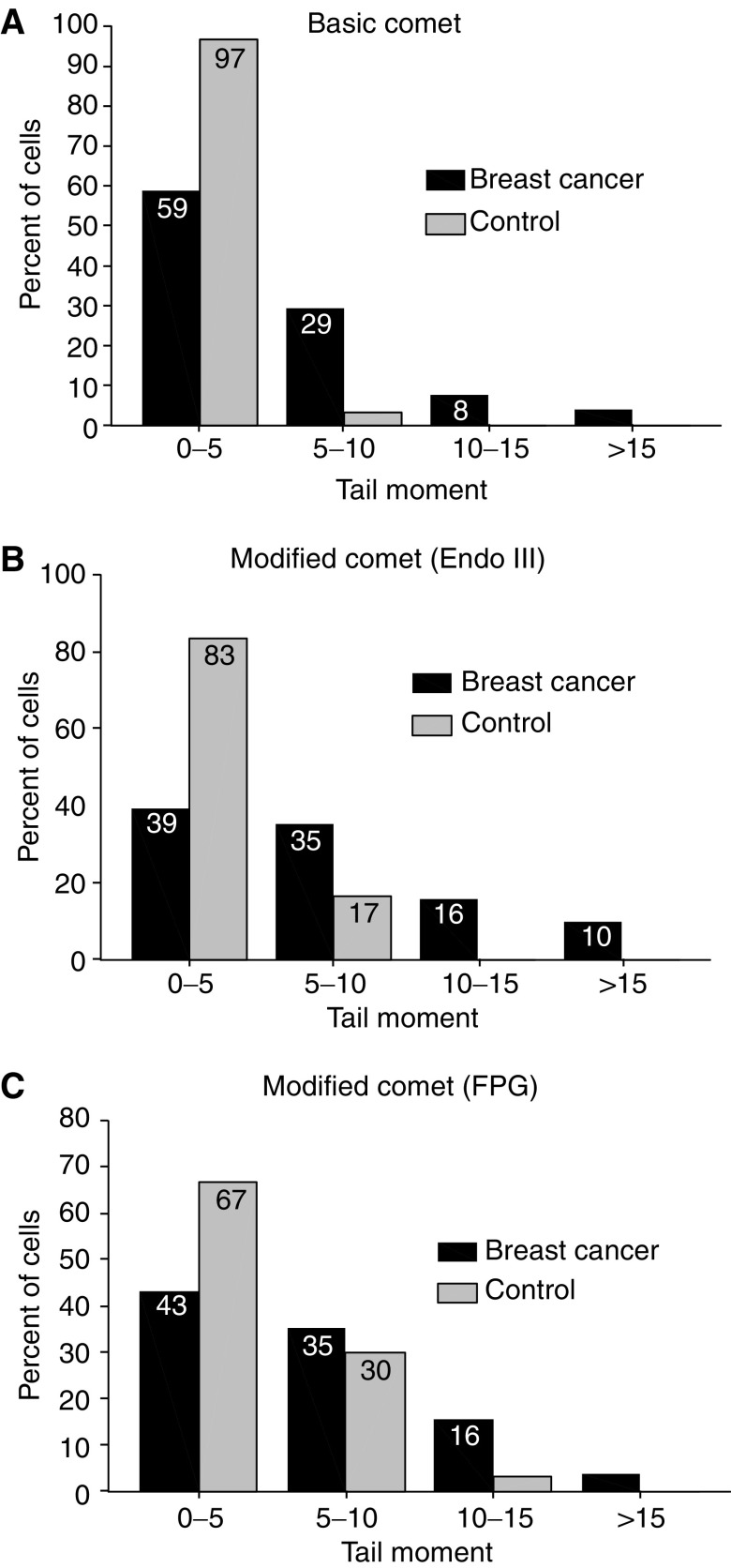
Frequency distribution of DNA damage in MNC of breast cancer and control patients. A greater percentage of the cells from the breast cancer patients had DNA damage levels in the highest damage categories of the frequency distributions. DNA damage expressed as tail moment=migrated DNA × tail length.

Figure 1 shows the overall frequency distribution of DNA damage in the MNC in all slides analysed for breast cancer and control patients. It shows that 97% of MNC from control patients had tail moment values of 0–5, and 3% of the cells showed tail moment values of 5–10. On the other hand, mononuclear cells from breast cancer patients had a larger percentage of their cells in the higher tail moment categories, indicating more DNA damage (59% of these cells showed tail moment value of 0–5, 29% showed tail moment values of 5–10, 8% showed tail moment of 10–15 and 4% of the cells showed tail moment values of >15). A similar pattern was observed in samples processed by the modified comet assays, with a larger percentage of cells showing higher tail moment values in breast cancer than control patients.

Tail moment of RJK853 internal control cells was not skewed and was not log transformed. The mean (s.d.) tail moment of internal control cells detected by basic comet assay was 1.7 (1.2) and the coefficient of variation (CV) was 0.71. The mean (s.d.) tail moment of internal control cells detected by modified comet assay (Endonuclease III) was 3.5 (2.9) and the CV was 0.82, and for FPG enzyme was 4.8 (3.7) and the CV was 0.77. This shows that the CV was (0.71–0.82) and demonstrated that the technique is highly reproducible. It also proved good laboratory practice and evidence for the ability to compare breast cancer and control patients as the DNA damage induced by the technique of comet assay itself was minimal.

#### Correlation analysis

Correlation analysis was performed between plasma homocysteine or RCF status of all patients (including breast cancer and control patients) and DNA damage levels in MNC of all patients at the log scale (using Pearson Correlation).

There was a significant negative correlation between RCF values and DNA damage detected by the modified comet assay using FPG enzyme. There was no significant correlation between plasma homocysteine of all patients and DNA damage (Table 4).

#### Impact of alcohol intake on DNA damage and folate status

Patients who consumed alcohol (total 52 patients, 38 breast cancer and 14 control patients) were divided into two groups according to their folate status. For the purpose of comparison, folate status was considered to be low if the RCF level was less than the median value (363.98 ng ml^−1^), and folate status high if the RCF was higher than the median value. Pearson Correlation was performed between log tail moment and log alcohol intake. In breast cancer patients who had low RCF values, there was a significant positive correlation between log alcohol intake and log tail moment detected by the modified comet assay (FPG) (Pearson Correlation Coefficient=0.58, *P*=0.04). In control patients who had low RCF values, there was no significant correlation between alcohol intake and DNA damage (Table 5).

In breast cancer patients who had high RCF values, there was a significant positive correlation between log alcohol intake and log tail moment detected by the modified comet assay (Endo III) (Pearson Correlation Coefficient=0.67, *P*=0.01) and log tail moment detected by the modified comet assay (FPG) (Pearson Correlation Coefficient=0.58, *P*=0.04). In control patients who had high RCF values, there was no significant correlation between alcohol intake and DNA damage (Table 5).

There was no significant correlation between log alcohol intake and log RCF in breast cancer patients (Pearson Correlation coefficient *r*^2^=0.05, *P*=0.73) or in control patients (Pearson Correlation Coefficient *r*^2^=−0.25, *P*=0.37).

## DISCUSSION

This study aimed to compare folate status and levels of DNA damage between breast cancer and benign breast disease (control) patients. Folate status was evaluated using RCF and plasma homocysteine, and DNA damage in the MNC was evaluated using the comet assay. Breast cancer patients tended to have a reduced folate status, that is, lower RCF and higher plasma homocysteine levels than control patients, but this failed to reach statistical significance. DNA damage in MNC of breast cancer patients was significantly higher than that of the control patients. Red cell folate was negatively correlated with DNA damage detected by the modified comet assay (using FPG enzyme). Alcohol intake was positively correlated (independent of folate status) with the level of DNA damage detected by the modified comet assay in breast cancer, but not the control patients. We were unable to demonstrate an interaction between folate status and alcohol intake in breast cancer or control patients.

The most frequently selected indicator of folate status is the erythrocyte folate level. Other indicators include serum and plasma folate levels (Jacques *et al*, 1993). The main limitation of serum and plasma folate levels is that they reflect transient changes in folate intake. The circulating folate concentration may be reduced in situations where there is no alteration in the overall status such as acute alcohol ingestion (Jacques *et al*, 1993). Folate is taken up only by the developing erythrocyte in the bone marrow, and therefore RCF is a good indicator of the long-term folate status (WU *et al*, 1975). Plasma homocysteine concentration increases when inadequate quantities of folate are available for the remethylation of homocysteine to methionine. Therefore, it is used as another indicator of folate status (Selhub *et al*, 1993). Although the current pilot study did not demonstrate a significant difference in RCF and plasma homocysteine concentrations, there is a possibility that a true difference might exist but was not demonstrated because of the small sample size in the study.

There is lack of data studying RCF and plasma homocysteine concentrations in breast cancer patients. One study investigated the incidence of breast cancer and the prediagnostic serum level of folate and homocysteine using serum specimens from the Washington County serum bank donated between 1974 and 1989 (Wu *et al*, 1999). Breast cancer cases (195 patients) were identified and were matched to 195 control subjects. There was no evidence for an association between serum folate or homocysteine levels and risk of breast cancer. There was no significant difference between the odds ratio of breast cancer for patients in the highest quartile of serum folate and that for patients in the lowest quartile. However, only nonfasting blood samples were available, which is known to affect the serum folate and homocysteine levels, and there are possible adverse effects of storage on the stored samples (Wu *et al*, 1999). A study recently reported by Beilby *et al* (2004) evaluated the folate status and the methylenetetrahydrofolate reductase (MTHFR) genotype of 141 breast cancer patients and 109 age-matched controls. The authors reported that serum folate was significantly lower in cancer patients and that the increased serum concentration of folate due to MTHFR polymorphism was associated with reduced risk of breast cancer (Beilby *et al*, 2004). These two studies used serum folate, in contrast to our study which used RCF, as a marker of the folate status.

In the study being reported in this paper, the alkaline comet assay was used to investigate the level of DNA damage in MNC. This was significantly higher in breast cancer patients compared to the controls. We used the modified alkaline comet assay described by Collins *et al* (1993) to increase the sensitivity of the assay by additionally measuring the oxidised pyrimidine or purine bases. The Endo III and the FPG enzymes recognize oxidatively damaged pyrimidines and purines, respectively, and nick the DNA at these sites creating single-strand breaks, which can be detected by the comet assay, thus increasing the sensitivity of the assay (Collins *et al*, 1993). In this study, the levels of DNA damage detected by basic and modified comet assay were significantly higher in breast cancer patients than controls. We studied the effect of folate status on DNA damage by correlation analysis. The negative correlation was only significant for the DNA damage detected by FPG enzyme, which demonstrate the significance of increasing the sensitivity of the assay. The study conducted *in vitro* by Duthie *et al* (2002) reported that folate deficiency increased DNA damage in cultured human lymphocytes and the DNA instability was inversely related to the concentration of folic acid available to the cells, and also demonstrated the sensitivity of the modified comet assay. These findings, as well as our result may indicate that the intake of folate adequate for prevention of clinical deficiency may not be optimal for maintaining DNA stability.

Another study has investigated the level of DNA damage, using the basic comet assay, in leucocytes of 88 breast cancer patients and 121 healthy controls as well as in 188 first-degree female relatives of breast cancer patients (Rajeswari *et al*, 2000). The authors reported that the maximum leucocyte DNA damage was observed in the breast cancer patients. The DNA damage in the leucocytes of the first-degree female relatives was 2.5 times higher compared to that of the healthy controls. The authors used the mean comet tail length (*μ*m) as a measure of DNA damage, in contrast to this present study which used the mean tail moment (migrated DNA × tail length), so direct comparison of the results is not possible (Rajeswari *et al*, 2000). However, the previous study showed that the DNA damage was significantly higher in leucocytes from breast cancer patients compared to controls, which agrees with our findings.

In the current study, there was no significant association between alcohol intake and breast cancer risk, possibly because a large proportion of women did not consume alcohol, the relatively low-alcohol consumption and the relatively small number of patients in the study. Our results agree with several other studies which have reported lack of association of breast cancer with low-to-moderate alcohol consumption (Nasca *et al*, 1990; Howe *et al*, 1991; Sneyd *et al*, 1991; Longnecker *et al*, 1995; Swanson *et al*, 1997; Stoll, 1999; Rohan *et al*, 2000; Kropp *et al*, 2001). In the case–control study conducted by Kropp *et al* (2001) in Germany, which included 706 premenopausal breast cancer patients and 1381 controls, provided evidence that low-to-moderate level of alcohol consumption does not increase breast cancer risk. However, there was a significantly increased risk of breast cancer for alcohol intake of more than 31 g day^−1^.

Several investigators have examined the association between total folate intake and breast cancer risk by levels of alcohol consumption (Zhang *et al*, 1999; Rohan *et al*, 2000; Sellers *et al*, 2001). The Nurses Health Study demonstrated a significant positive association between alcohol intake and breast cancer risk, with a 24% increased risk among women consuming at least 15 g day^−1^ alcohol compared to nondrinkers (Zhang *et al*, 1999). Another cohort study from the Mayo Clinic, conducted by Sellers *et al* (2001) reported that women with low dietary folate (less than 186 *μ*g day^−1^) and alcohol intake, more than 4 g day^−1^, had a 59% increased risk of breast cancer. The cohort study conducted by Rohan *et al* (2000) reported that in women who consumed more than 14 g day^−1^ of alcohol, women with low folate intake (less than 224 *μ*g day^−1^) had a higher risk of breast cancer compared to women with high folate intake (more than 354 *μ*g day^−1^). In the current study, patients who consumed alcohol were divided into low and high folate groups according to their RCF levels. There was a trend of a positive correlation between alcohol intake and DNA damage in breast cancer but not the control patients irrespective of their RCF status. This could be due to relatively low alcohol consumption and the small number of patients in this study.

In conclusion, DNA damage levels were found to be significantly higher in MNC of breast cancer patients compared to benign breast disease control patients. Breast cancer patients tended to have lower RCF and higher plasma homocysteine concentrations, but these differences were not statistically significant. There was evidence of a negative correlation between DNA damage and RCF in all patients. There was evidence of a positive correlation between DNA damage and alcohol intake in breast cancer, but not the control patients. However, this correlation was not affected by RCF status possibly as a result of the small numbers of patients in this study and relatively low alcohol consumption.

This is the first study to investigate the level of DNA damage and folate status in breast cancer and benign breast disease patients, providing some evidence that reduced folate may be implicated in the development of breast cancer.

## Figures and Tables

**Table 1:** Characteristics of breast cancer and control patients

	**Breast Cancer (s.d.)**	**Control (s.d.)**	**Significance (*t*-test)**
Mean age (years)	57 (14.3)	51 (14.1)	*P*=0.08
Weight (kg)	69.1 (12.8)	68.7 (11.6)	*P*=0.86
Height (m)	1.60 (0.05)	1.59 (0.06)	*P*=0.29
BMI (kg m^−2^)	26.6 (4.7)	27.1 (4.9)	*P*=0.67
			
*Menopausal status*			
Premenopausal	21	14	*P*=0.26^a^
Postmenopausal	43	16	
			
*Alcohol consumption* (*g day*^−1^)	3.49 (4.39)	4.69 (7.7)	*P*=0.34
			
*Smoking*			*P*=0.82^a^
Nonsmokers	55	27	
Smokers	9	3	

a *χ*^2^ test s.d.=standard deviation.

**Table 2:** Blood folate status measurements in breast cancer and control patients

**Variable**	**Breast cancer geometric mean (95% CI)**	**Controls geometric mean (95% CI)**	**Significance (Student's *t*-test)**
Red cell folate (ng ml^−1^)	339.07 (303.9–378.3)	379 (322.8–446.3)	*P*=0.246
Plasma homocysteine (*μ*mol l^−1^)	11.90 (10.61–13.25)	10.14 (9.01–11.43)	*P*=0.073

**Table 3:** Levels of DNA damage in mononuclear cells of breast cancer and control patients

**Variable**	**Breast cancer mean log TM (s.d.) [95% CI]**	**Control mean log TM (s.d.) [95% CI]**	**Significance (Student *t*-test)**
1. Basic alkaline comet	1.46 (0.66) [1.26–1.66]	−0.177 (0.79) [−0.43–0.08]	*P*<0.0001*
2. Modified comet (Endo III)	1.77 (0.70) [1.56–1.98]	0.86 (0.81) [0.59–1.13]	*P*<0.0001*
3. Modified comet (FPG)	1.67 (0.62) [1.46–1.88]	0.99 (0.94) [0.72–1.26]	*P*<0.0001*

* Significant (*t*-test). (s.d.)=standard deviation; 95% CI=95% confidence interval; TM=tail moment=migrated DNA × tail length; Endo III=endonuclease III enzyme (to detect oxidised pyrimidines); FPG=formamidopyrimidine glycosylase enzyme (to detect oxidised purines).

**Table 4:** Correlation between folate status and levels and DNA damage in mononuclear cells of all patients

	**Log mean tail moment**		
**Variable**	**Basic Comet Mean (s.d.) 0.82 (1.06)**	**Endo III Mean (s.d.) 1.39 (0.85)**	**FPG Mean (s.d.) 1.36 (0.82)**
*Red cell folate*			
Pearson Correlation (*r*^2^)	−0.155	−0.157	−0.26*
(significance)	(*P*=0.18)	(*P*=0.18)	(*P*=0.02)*
			
*Plasma homocysteine*			
Pearson Correlation (*r*^2^)	0.08	0.06	0.08
(significance)	(*P*=0.48)	(*P*=0.58)	(*P*=0.48)

* Significant (Pearson Correlation). TM=tail moment=migrated DNA × tail length; Endo III=endonuclease III enzyme; FPG=formamidopyrimidine glycosylase enzyme.

**Table 5:** Correlation between alcohol intake and DNA damage in mononuclear cells of breast cancer and control patients who had low and high red cell folate (RCF) values

	**Log mean tail moment**		
	**Basic Comet**	**Endo III**	**FPG**
	**Mean (s.d.)**	**Mean (s.d.)**	**Mean (s.d.)**
**Log alcohol intake (g day^−1^)**	**0.82 (1.06)**	**1.39 (0.85)**	**1.36 (0.82)**
*Low red cell folate*			
Breast cancer			
Pearson Correlation (*r*^2^)	0.25	0.36	0.58
(Significance)	(*P*=0.42)	(*P*=0.24)	(*P*=0.04)*
			
Controls			
Pearson Correlation (*r*^2^)	−0.32	−0.17	−0.57
(Significance)	(*P*=0.42)	(*P*=0.68)	(*P*=0.13)
			
*High RCF*			
Breast cancer			
Pearson Correlation (*r*^2^)	0.4	0.67	0.58
(Significance)	(*P*=0.19)	(*P*=0.01)*	(*P*=0.04)*
			
Controls			
Pearson Correlation (*r*^2^)	−0.007	−0.21	−0.75
(Significance)	(*P*=0.99)	(*P*=0.72)	(*P*=0.13)

* Significant (Pearson Correlation). Low red cell folate (RCF)=less than median RCF value=363.98 ng ml^−1^. High red cell folate (RCF)=more than median RCF value=363.98 ng ml^−1^.
